# Mesoscale assembly of chemically modified graphene into complex cellular networks

**DOI:** 10.1038/ncomms5328

**Published:** 2014-07-07

**Authors:** Suelen Barg, Felipe Macul Perez, Na Ni, Paula do Vale Pereira, Robert C. Maher, Esther Garcia-Tuñon, Salvador Eslava, Stefano Agnoli, Cecilia Mattevi, Eduardo Saiz

**Affiliations:** 1 Department of Materials, Centre for Advanced Structural Ceramics, Imperial College London, London SW7 2AZ, UK; 2 Department of Physics, Imperial College London, London SW7 2AZ, UK; 3 Department of Chemical Sciences, University of Padua, 35131 Padua, Italy

## Abstract

The widespread technological introduction of graphene beyond electronics rests on our ability to assemble this two-dimensional building block into three-dimensional structures for practical devices. To achieve this goal we need fabrication approaches that are able to provide an accurate control of chemistry and architecture from nano to macroscopic levels. Here, we describe a versatile technique to build ultralight (density ≥1 mg cm^−3^) cellular networks based on the use of soft templates and the controlled segregation of chemically modified graphene to liquid interfaces. These novel structures can be tuned for excellent conductivity; versatile mechanical response (elastic-brittle to elastomeric, reversible deformation, high energy absorption) and organic absorption capabilities (above 600 g per gram of material). The approach can be used to uncover the basic principles that will guide the design of practical devices that by combining unique mechanical and functional performance will generate new technological opportunities.

## Abstract (web summary)

Graphene’s properties are well known, but turning this atomically thin material into three-dimensional devices remains a challenge. Here, the authors report an assembly process for chemically modified graphene into three-dimensional cellular networks, with control over the physical properties of the resulting materials.

## Graphical abstract

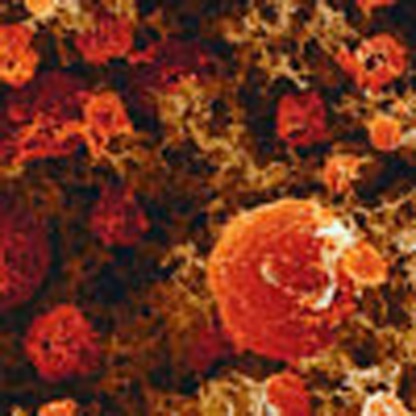

A wide range of emergent technologies, from tissue engineering to energy storage or environmental cleaning demand strong, lightweight porous structures able to provide a wide range of functionalities such as support for cells or nanoparticles or high thermal or electrical conductivity. The engineering and scientific challenge is to design and fabricate these structures in practical dimensions while maintaining an accurate control of their chemistry and architecture from the nano-level and up. This is particularly difficult when using a material like carbon. Although its low density and versatility make it extremely appealing in many applications, its reactivity and hydrophobicity creates serious difficulties for most conventional processing techniques. In this respect carbon nanomaterials such as nanotubes or graphene have not only opened new opportunities but also new challenges. For example, graphene is a truly two-dimensional material with unique functional and mechanical properties from tunable electrical and optical response to high intrinsic stiffness and strength, chemical versatility, controllable permeability or extremely high specific surface area (2,630 m^2^ g^−1^) (refs 1, 2, 3, 4). If properly integrated into macroscopic highly porous complex structures, graphene has the potential to form novel platforms for a wide range of functional systems from batteries to supercapacitors, reactive catalytic supports or filters and membranes to name a few^5^,^6^,^7^. However, to achieve this goal we need to develop ways for the controlled assembly of three-dimensional (3D) structures using a two-dimensional (2D) building block.

As the first reports on graphene 3D structures based on chemical vapour deposition coatings on metallic foams, different approaches based on freeze drying and subcritical drying^8^,^9^, leavening^7^, nucleation boiling^10^, hydrothermal processes^11^,^12^, hydrothermal carbonization^13^ or microwave irradiation^14^ have been used to fabricate porous 3D graphene and graphene-based networks. However, the challenge still remains on how to effectively tailor their chemistry and architecture for specific applications. Furthermore, structural design has to be based on a fundamental understanding of how architecture determines performance.

These challenges underline the need to develop more flexible and cost effective processing technologies that allow the manipulation of structure. In this respect, wet-processing approaches such as emulsion-based techniques are very appealing. They are well developed for a wide range of organic and inorganic materials and they are very attractive due to their flexibility, scalability and relative low cost^15^,^16^,^17^. Wet-processing with graphene still requires much research as it depends on an accurate control of surface chemistry and particle size to enable the formulation of suspensions with controlled rheological response. Here we show that the use of chemically modified graphene (CMG, namely graphene oxide, GO, and its reduced form rGO) opens many possibilities.

In this paper, we propose a self-assembly strategy for the fabrication of complex CMG cellular networks (CMG-CNs) via a multi-step soft/hard template mechanism that combines emulsion and ice templating. Our approach is based on controlling the segregation of CMG to liquid interfaces to use it both as an emulsifier and as a building block. Two key challenges are maintaining the stability of the structures and manipulating the properties of CMG such that they approach those of pristine graphene. To this purpose, a range of additives and thermal treatments are used. This approach allows the integration of 2D CMG into macroscopic 3D cellular networks with tunable hierarchical structures and performance. As a result we can prepare materials with excellent electrical conductivity, high mechanical strength, recoverable structural deformation, high levels of energy absorption or high organics adsorption capabilities.

## Results

### Assembling 3D CMG-CNs with controlled architectures

**Figure 1:**
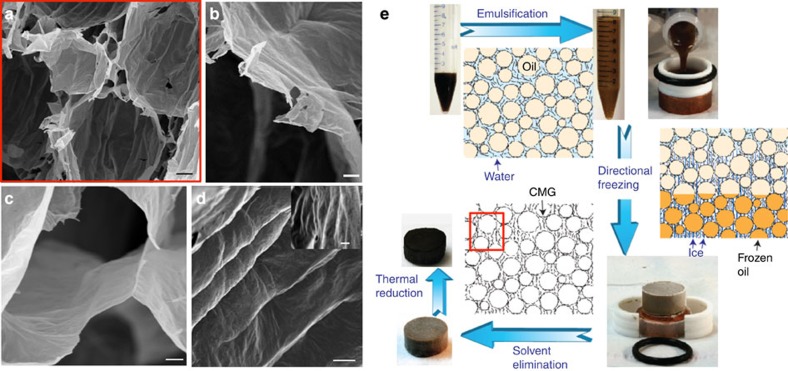
Microstructural architecture and assembly strategy of CMG-CNs. (**a**) Overview of the CMG-CN architecture, composed of nearly spherical pores in the micrometre scale, designed by the emulsion droplet templates. Thin CMG self-assembly: at the pore walls as a result of their entrapment at the interface between water and oil droplets during emulsification (**b**) and at the triple junction between adjacent cells, whose arrangement is template by ice (**c**). Cell walls surface micro to nanorugosity patterned by the ice crystals during unidirectional freezing (**d**). SEM images of 5.6 mg cm^−3^ GO-CN (**a**–**d**) that after thermal reduction at 1,000 °C results in rGO-CN of 2.2 mg cm^−3^. (**e**) Assembly strategy of CMG-CNs and their structural evolution from emulsification, unidirectional freezing to freeze drying. Following the arrows: As-prepared aqueous GO suspensions (GO-sus) are emulsified with 75 vol% of the hydrophobic internal phase (toluene) resulting in GO emulsions (GO-em) composed by oil droplets dispersed in the GO aqueous continuous phase. GO flakes act as a surface-active amphiphile self-assembling at the oil/water interface. GO-em are moulded into cylindrical shaped moulds and subsequently directionally frozen. As unidirectional freezing of GO-em progresses the ice crystals in the water phase encapsulate liquid oil droplets (as their solidification temperature is much lower) templating the roughness of CMG at the droplet wall. After eliminating the solvents during freeze drying GO-CNs are obtained with the ice and emulsion droplet templating the cellular architecture (**a**–**d**). rGO-CN are obtained after thermal annealing. Scale bars, 10 μm (**a**), 2 μm (**b**), 1 μm (**c**) and 2 μm (**d**).

The assembly strategy (Fig. 1e) is based on the preparation of oil-in-water (o/w) GO emulsions (GO-em), by the emulsification of water-based GO suspensions (GO-sus) with a hydrophobic (oil) phase. GO acts as a surface-active amphiphile^18^ self-assembling at the interface between the oil droplets and the water phase and stabilizing the GO-em for several months. The oil droplets template the formation of spherical- to polyhedral-shaped cells (Fig. 1a). The cell walls and the volume between cells are formed by an arrangement of CMG flakes (Fig. 1b,c). The challenge is to prepare stable emulsions with high oil content to fabricate highly porous materials while maintaining the stability of the network once the liquid phase is removed.

It has been previously shown that GO can act as a colloidal emulsifier with its interfacial activity being very dependent on pH due to its structural configuration of more hydrophobic basal plane and more hydrophilic plane edges^18^. However, the use of emulsions to prepare porous materials requires enough GO segregation to the water–oil interface to create stable emulsions with high oil content and controllable droplet sizes in the low micrometre range and, at the same time, to achieve highly concentrated GO suspensions in water such that the structure withstands the capillary forces during solvent elimination. To this end, we have taken advantage of the pH-dependant interfacial activity of GO and have designed a two-step process. First, we prepare stable, highly concentrated GO suspensions in water at neutral pH to assure a high degree of deprotonated –OH and –COOH groups on the surface of GO and to achieve therefore a good dispersion^19^. Just before emulsification, the suspension pH is adjusted between 2 and 3. At such acidic conditions, the oxygen functional groups on GO surface are less deprotonated^18^. This reduces the hydrophilicity of the basal plane and the flakes preferentially segregate to the o/w interfaces (Supplementary Fig. 1). To enable more flexibility in the process, diverse organic additives can be used to enhance dispersion and emulsification ability.

**Figure 2:**
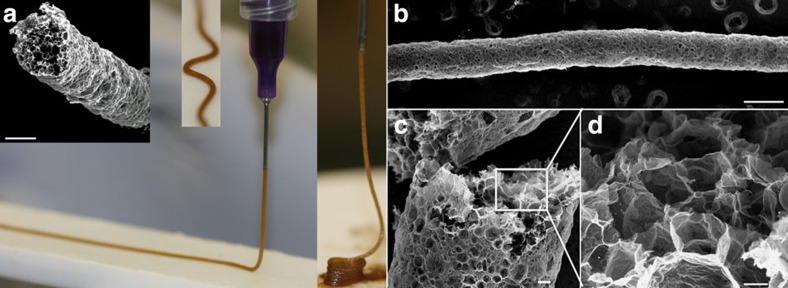
CMG-CN wires. The viscoelastic properties of the GO emulsion system developed in this work enables its extrusion through micro needles resulting in GO emulsion wires that maintain their shape (straight, curved or spirals) (**a**) and can be further processed by the approach described in this paper. In (**b**–**d**) details of rGO-CN wire and internal cellular microstructure after thermal treatment at 1,000 °C in Ar/H_2_ atmosphere. GO emulsions prepared by the emulsification of 65 vol% decane in 1 wt% GO suspensions containing 1.2 wt% organic additives (1:1, PVA:sucrose). The wires are several centimeters long and down to 200 μm in diameter. Scale bars, 200 μm (**a**), 300 μm (**b**), 20 μm (**c**) and 10 μm (**d**).

With our approach we are able to homogeneously disperse high concentrations of GO in water (>10 mg ml^−1^) and subsequently maximize the surface activity of the GO flakes (by means of suspension pH and/or additives) leading to stable emulsions with up to 75 vol% of the oil phase and droplet sizes in the low micrometre scale, one order of magnitude smaller than that previously reported for GO^18^. The viscoelastic properties of the emulsions can be controlled by adjusting the concentration of GO in the continuous phase or the concentration of oil, allowing the fabrication of coatings or the extrusion of long wires that can be down to 200 μm in diameter (Fig. 2). This opens new opportunities for the fabrication of graphene-based complex structures at the macroscale. For example, this technique can be explored for the design of inks with rheological properties tailored for different wet-processing techniques to build structures with porosity determined by droplet size, shape and concentration in the emulsion.

To maintain the stability of the porous structure after removing the liquid phase while effectively manipulating the nano to micro architecture of the network, we use a combination of ice templating and freeze drying. After emulsification, the GO-em is directionally frozen and subsequently the solvents are eliminated by freeze drying (Fig. 1e). As directional freezing progresses, the growing ice crystals control the alignment of CMG in the water phase (and consequently the architecture of the triple junctions between adjacent cells (Fig. 1c)) and encapsulate the liquid oil droplets (as the oil solidification temperature is much lower than that of water). As the droplets are still ‘soft’ in the liquid phase, the roughness of the ice templates the formation of micro to nanorugosity on the CMG-CNs cell walls (Fig. 1d).

In contrast to the lamellar structures resulted from the traditional freeze casting of aqueous suspensions^17^ here the structural evolution is more complex. The emulsion templates the formation of spherical to polyhedral cells to create a cellular network (Fig. 1a) with ice forming between the oil droplets. In the emulsions used to fabricate cellular networks the high concentration of oil droplets may impede the full formation of lamellar ice (as it has been observed with highly concentrated particle suspensions)^20^.

**Figure 3:**
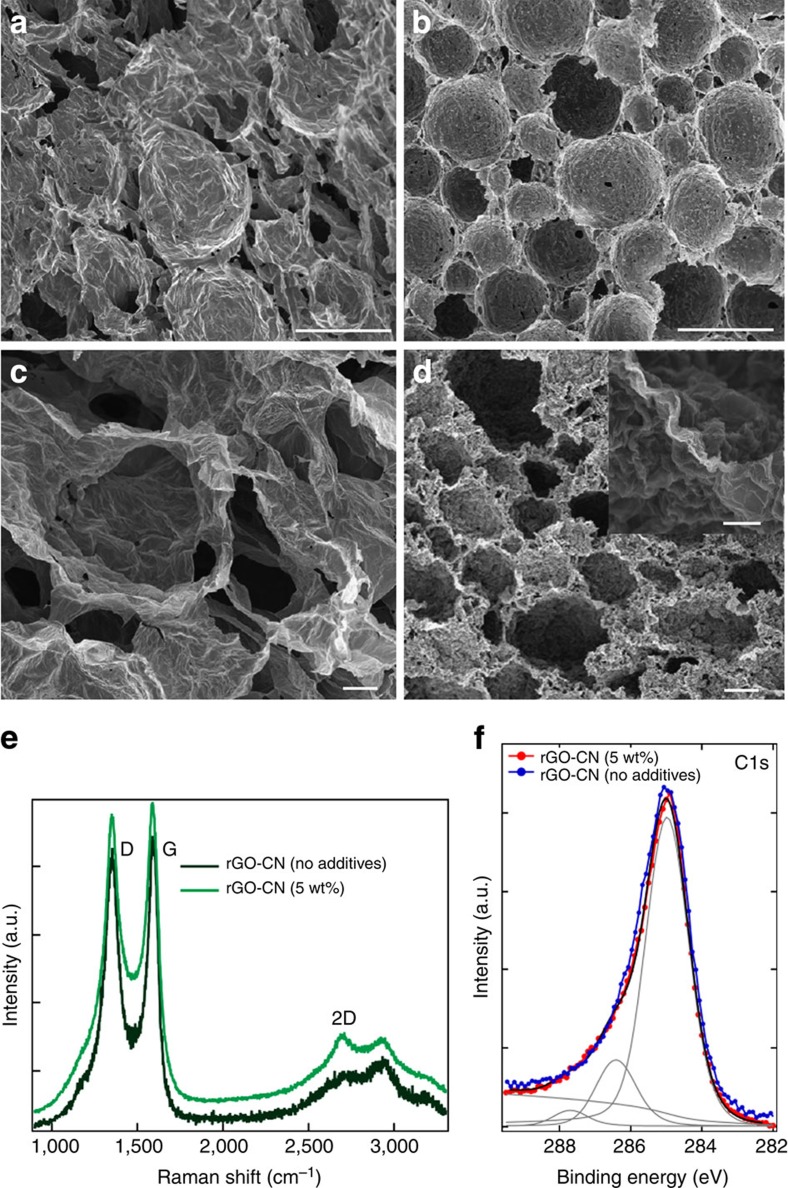
Effect of additives on CMG-CNs microstructure and crystallinity. SEM images of: GO-CNs produced without (3.3 mg cm^−3^ density, 65 μm average cell size) (**a**) and with the addition of 5 wt% organic additives (PVA:sucrose, 1:1 wt%) in 0.65 wt% GO-sus (15 mg cm^−3^ density, 40 μm average cell size) (**b**); rGO-CNs thermal treated at 1,000 °C in Ar/H_2_ atmosphere produced without (1 mg cm^−3^ density, 63 μm average cell size) (**c**) and with the addition of 5 wt% organic additives (112 mg cm^−3^ density, 7 μm average cell size) (**d**). Shrinkage during the thermal treatment results in highly wrinkled rGO at the cell walls (**d**). The corresponding Raman spectra (514 nm laser) and C1s XPS of rGO-CNs (prepared without and with organic additives (SEM in **c**,**d**)) are represented in **e** and **f**, respectively. The letter ‘D’ and ‘G’ stand for two characteristic Raman active modes for graphene and other carbon allotropes and the D/G ratio is a measure of the density of defects present in the carbon material^28^. The C1s XPS spectra (h*ν*=1,253.6 eV) collected on rGO-CN with 5 wt% additives is indicated in red colour whereas the rGO-CN produced without organic additives appears in blue colour. The C1s spectrum collected on rGO-CN with 5 wt% additives was fit by Doniach–Sunjic function after subtracting a Shirley background as indicated in the lowermost spectrum. The different components related to various chemical shifts of carbon bonds are indicated. The component at 284.6 eV is due to the sp^2^ carbon, the peak at 286.5 eV is related to the remaining C–O bonding and possible carbon sp^3^ defects, and the component at 287.8 eV is related to residual C=O bonding^24^. The full width at half maximum (FWHM) for the C1s core level for rGO-CNs, with initially 5 wt% additives is narrower than for rGO-CNs without additives. This reflects the different sp^2^ content, which is about 82% in rGO-CNs, with initially 5 wt% additives (before reduction) and about 80% in rGO-CNs, produced without additives. Scale bars, 100 μm (**a**,**b**), 10 μm (**c**,**d**) and 2 μm (insert image in **d**). a.u., arbitrary unit.

This processing approach can be used to form highly porous GO-CNs with densities ranging from 2 to 20 mg cm^−3^. The cell sizes are in the range 20–65 μm and the walls are formed by a complex arrangement of CMG (Fig. 1a–d). The cell size can be mainly controlled varying the amount and size of GO flakes and emulsification energy, whereas the density can be tuned through the amount of GO in suspension and oil concentration. Different organic additives (such as sucrose or polyvinyl alcohol (PVA)) can be added to further control the architecture and properties although it is possible to fabricate stable structures without the use of any additive. For example, PVA molecules adsorbed on the surface of GO^21^,^22^ can change its wettability (Supplementary Fig. 2). This can further boost the surface activity of GO, as evidenced by the improvement in emulsification ability (Supplementary Fig. 3a) and the stabilization of smaller droplets that will template smaller cells (Supplementary Fig. 3d). On the other hand, sucrose acts as a binder that helps to hold the structure together after removing the liquids and also affects the shape of the ice crystals formed during templating^23^. As a result, it determines the topography of the cell walls forming patterns that can go from straight (Supplementary Fig. 4a–b) to circular (Supplementary Fig. 4c,d). Organic additives can further be used to manipulate the formation of cell windows (or openings) of controlled shape at the micro scale (detailed information in Supplementary Fig. 4). Before thermal reduction, a typical additive-free GO-CN (Fig. 3 and Supplementary Fig. 4) has a density of the order of 2.5–3.5 mg cm^−3^ with an average cell size of 65 μm. By adding up to 5 wt% organic additives (PVA:sucrose, 1:1 in wt%) denser GO-CNs (up to 20 mg cm^−3^) with smaller, spherical shaped cells (up to 40 μm in average size) are formed (Fig. 3a,b and Supplementary Fig. 3c,d).

The final step is a thermal treatment in a reducing atmosphere to promote the reduction of GO into rGO with the decomposition of functional groups^24^ and organic additives resulting in mass loss and shrinkage (Supplementary Fig. 3c, Supplementary Figs 6 and 7). Reduction has been performed at temperatures ranging from 300 up to 2,400 °C under 90% Ar/10% H_2_ or in vacuum (25–60 Pa) in a graphite furnace. During this process, the degree of densification and consequently the nano to macrostructural features of the rGO-CNs including its crystallinity can be further manipulated as the reduction conditions (time, temperature and atmosphere) and the additives will determine the evolution of the structure. When the GO-CNs are thermally treated at 1,000 °C, the linear dependency of mass loss versus GO content (Supplementary Fig. 5) suggests that 91% of the organics and 64% of the GO mass are eliminated. Thermal annealing at higher temperatures in a graphite furnace is likely to be accompanied by a further elimination of residual functional groups on GO and amorphous carbon as indicated by the slight increase in mass loss (Supplementary Fig. 6).

**Figure 4:**
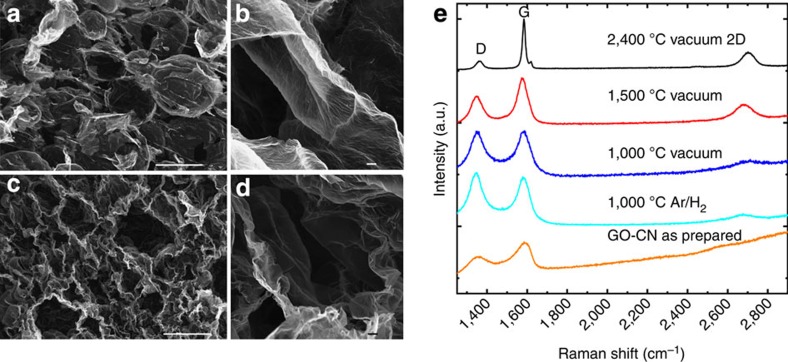
Effect of the thermal treatment temperature and atmosphere on rGO-CNs microstructure and crystallinity. SEM images of rGO-CNs thermally treated in a graphite furnace under high vacuum at: 1,000 °C (**a**,**b**) (1.4 mg cm^−3^ density) and 2,400 °C (**c**,**d**) (7.2 mg cm^−3^ density). The density of as-prepared GO-CNs (0.65 wt% GO and 0.3 wt% additives (PVA:sucrose in 1:1 wt%) in suspension) before thermal treatment is 2.9–3.3 mg cm^−3^. rGO-CNs reduced at 2,400 °C present highly wrinkled cell walls (**c**,**d**) as a result of the high degree of shrinkage (87%) during thermal treatment. (**e**) Raman spectra of the CMG-CNs as prepared and under different thermal treatments in Ar/H_2_ atmosphere or in vacuum in a graphite furnace. The Raman spectrums were obtained using 514 nm laser. The letter ‘D’ and ‘G’ stand for two characteristic Raman active modes for graphene and other carbon allotropes and the D/G ratio is a measure of the density of defects present in the carbon material^28^. Scale bars, 100 μm (**a**–**c**) and 2 μm (**b**–**d**). a.u., arbitrary unit.

rGO-CNs resulted from thermally reducing an additive-free GO-CN at 1,000 °C in Ar/H_2_ are ultralight, with densities as low as 1 mg cm^−3^ (99.95% porosity) and average cell size of 63 μm. They are 70% lighter but with a cell size very similar to the starting non-reduced material as a result of the loss of chemical groups attached to GO during firing (Fig. 3a,c and Supplementary Fig. 3b–d). This material is lighter than the lightest silica aerogels^25^ and as light as the lightest Ni microlattice^26^. On the other side, when adding up to 5 wt% additives (PVA:sucrose, 1:1 in wt%), and under the same emulsification and thermal treatment conditions, the network undergoes severe densification resulting in a density >100 mg cm^−3^ and average cell size of 7 μm (Fig. 3b,d and Supplementary Fig. 3b–d). This corresponds to a density increase of over 700% with cells that are 87% smaller after reduction. In this case, the melting of sucrose to form high-molecular-weight compounds, ‘caramel’, during its decomposition triggers a process akin to ‘liquid-phase sintering.’ As sucrose melts the liquid flows within the cellular network due to the action of capillary forces bonding the neighbouring rGO flakes (Supplementary Fig. 3b–d). The process enables the formation of CMG-CN structures with a wide range of final densities that go over two orders of magnitude from 1 to over 200 mg cm^−3^, cell sizes (around one order of magnitude between 7 and 65 microns) and shape (spherical to polyhedral) (Figs 3 and 4, Supplementary Fig. 4). It is also possible to manipulate the cell wall roughness and interconnectivity from nano to micro levels (Figs 1d, 3 and 4, Supplementary Fig. 5).

**Figure 5:**
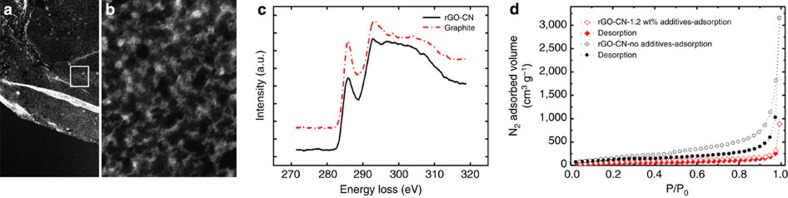
STEM, EELS and N_2_ adsorption isotherms for ultralight rGO-CNs. (**a**,**b**) STEM HAADF images of an rGO-CNs cell wall. The pores appear in darker contrast on the brighter background of the rGO-CNs. High magnification image (from the region indicated in **a** reveals mainly mesopores with sizes ranging between ~2 and 10 nm (**b**). The nanoporosity decorates the rGO-CN restored sp^2^ network as shown by EELS characterization for rGO-CNs and graphite (**c**). By comparing the K near-edge structure of both materials, a fraction of 95% of sp^2^ bonding is found (**d**). N_2_ adsorption curve for rGO-CN with 431 m^2^ g^−1^ SSA (structure prepared with no additives) and 180 m^2^ g^−1^ SSA (structure prepared with 1.2 wt% additives). Scale bars, 0.2 μm (**a**) and 10 nm (**b**). a.u., arbitrary unit.

Further, the elimination of functional groups upon thermal reduction (~65 to 90% of the mass is lost during thermal treatment depending on the rGO-CNs composition, Supplementary Fig. 5) generates nanopores and defects on the CMG-CNs walls, revealed in scanning transmission electron microscopy (STEM) high-angle annular dark field (Fig. 5a,b). After a thermal treatment at 1,000 °C in Ar/H_2_, there is a wide distribution of pores on the rGO-CNs walls, (pore size data obtained by nitrogen adsorption, in the range 2–100 nm is presented in Supplementary Fig. 7). The nanoporosity decorates the restored sp^2^ network on the rGO-CN cell walls as revealed by electron energy loss spectroscopy (Fig. 5c). By comparing the K near-edge structure of rGO-CN with graphite^27^, we identify the presence of a sp^2^ component nearly close to 95%. The nitrogen adsorption curves of rGO-CNs correspond to a type III isotherm with a small high-pressure hysteresis that is typical for macro and mesoporous materials (Fig. 5d). Ultralight additive-free rGO-CNs with a density of 1 mg cm^−3^ reach a specific surface area of 422±10 m^2^ g^−1^. The addition of additives (1.2 wt%) leads to a reduction in the accessible surface of the networks down to 170±10 m^2^ g^−1^. This can be attributed to the densification of the CMG network that accompanies the elimination of organic additives during thermal treatment (Supplementary Fig. 3b,c).

The Raman spectra of rGO-CNs confirm the formation of predominantly crystalline rGO upon thermal reduction (Figs 3e and 4e). Furthermore, the rGO-CNs produced with organic additives present a more pronounced 2D peak at around 2,700 cm^−1^, than rGO-CN produced without any additive (Fig. 3e). This indicates that the presence of carbon (due to organics decomposition) during thermal reduction promotes the recrystallization of graphene.

XPS characterization reveals that the oxygen content is about 4% for rGO-CNs prepared initially with 5 wt% additives (in GO-sus) after reduction at 1,000 °C and progressively decreases for higher annealing temperatures. Interestingly, the sp^2^ fraction of rGO-CNs produced with 5 wt% additives is slightly higher (82%) than for additive-free rGO-CNs (80%) as revealed by XPS (Fig. 3f). This further supports the Raman evidence that the presence of the organic additives as a carbon source favours restoration of sp^2^ bonding.

Furthermore, the crystalline quality of CMG can be greatly improved with further treatments at temperatures above 1,000 °C (Fig. 4e). The D/G intensity ratio for GO is about 0.62, whereas the ratio is 1.1 after annealing at 1,000 °C in Ar/H_2_ and 0.96 for thermal treatment in a graphite furnace (under vacuum) at the same temperature. The increase of the D/G peak intensity ratio for initial annealing at 1,000 °C in Ar/H_2_ and the decrease after annealing in a graphite furnace can be interpreted as a continuous decrease of the defect density as the evolution of D/G intensity ratio with defects can drastically change depending on the level of defects density^28^. Thermal annealing in the graphite furnace provides a carbon source that favours restoration of the sp^2^ bonding similar to the effect of organic additives (Fig. 3e). The D/G intensity decreases with increasing annealing temperature reaching a value of 0.16 after annealing at 2,400 °C. This value is comparable to mildly defected graphene with single and double vacancies^28^, suggesting that the sp^2^ network has been restored and lattice defects are now sparse with an average distance of about 25 nm. At the same time the intensity of the 2D peak is progressively recovered with the thermal treatment. Although in GO the 2D peak is barely detectable^24^ (Fig. 4e), the 2D/G intensity ratio increases with annealing temperature from 0.11 to 0.34 after heating up to 1,500 °C. After annealing at 2,400 °C the full width at half maximum of the 2D peak is about 67 cm^−1^ and the 2D/G intensity ratio about 0.3 suggesting that the material now consists of few misoriented graphene layers^29^. The slight decrease in 2D/G peak between 1,500 °C (0.34) and 2,400 °C (0.30) is likely to be due to the high densification of the materials leading to a closer proximity between flakes (wrinkled rGO-CN cell walls, Fig. 4d). These results are comparable to those obtained after applying high temperature (1,500 °C) and pressure (20 MPa) to CMG films^30^. They open new possibilities for the fabrication of hierarchical 3D structures with a restored sp^2^ network.

### Mechanical behaviour and electrical conductivity of rGO-CNs

**Figure 6:**
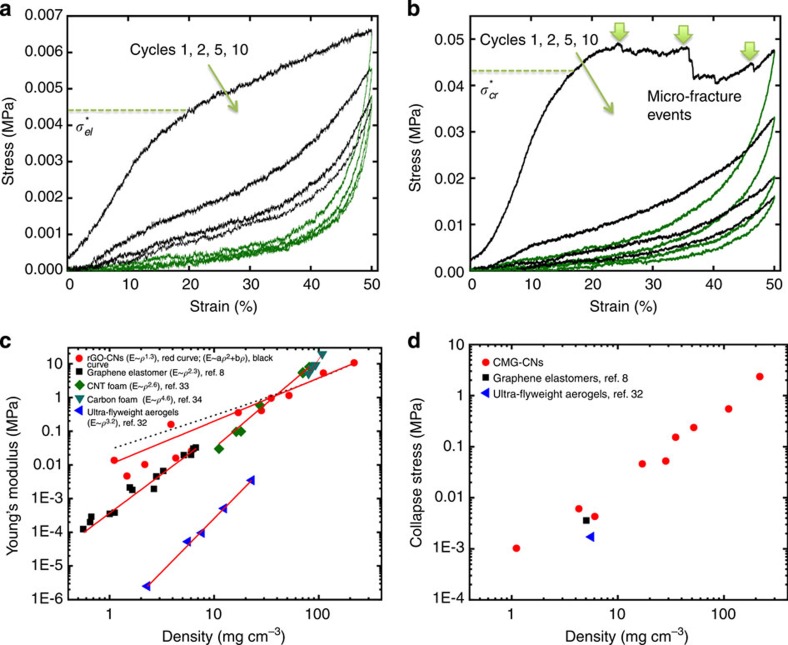
Multicycle compressive properties of rGO-CNs and their properties as a function of density. (**a**) Stress-strain curves of rGO-CN (6.1 mg cm^−3^) showing 98 and 95% recoverable deformation after one and 10 cycles of compression, respectively. rGO-CN was produced with the addition of 1.2 wt% additives (PVA:sucrose 1:1 wt%) to the GO suspension and thermally treated at 300 °C in Ar/H_2_. (**b**) Stress-strain curves of an rGO-CN with 17 mg cm^−3^ showing a plateau characteristic for micro-fracture events similar to those observed in the compression of elastic-brittle foams. This material shows 98 and 94% recoverable deformation after the first and tenth cycles of compression, respectively. The rGO-CN was produced with the addition of 2.5 wt% additives (PVA:sucrose 1:1 wt%) to the GO suspension and thermally treated at 1,000 °C in Ar/H_2_. (**c**) Young’s modulus E and (**d**) collapse stress versus density *ρ* for rGO-CNs and several low-density carbon-based porous materials reported in literature. An empirical power law fitting (red line) results in an exponent that is significantly lower (1.3) than those reported for other porous carbon structures (2.3–4.6) and the quadratic dependence expected for an open cell. The data have also been fitted to the *E*=a*ρ*^2^+b*ρ* equation resulting from the theoretical analysis of Gibson and Ashby^35^ (black dashed line) for a closed-cell foam. The fittings suggest that the rGO-CNs behaviour is closer to a closed-cell system where the cell wall membrane stresses have a significant role. The criteria used to calculate the collapse stresses is shown in **a** and **b**. 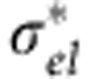 represents the elastic collapse stress (characteristic for lighter materials) and 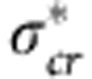 the brittle collapse stress characteristic for denser rGO-CNs.

The mechanical response under compression reflects the range of architectures that can be fabricated using this approach. Their behaviour changes from elastomeric for the lighter materials to elastic-brittle for the denser rGO-CNs. In all cases, during the first compression cycle the structures exhibit first a predominantly linear elastic region that can be associated to cell wall bending and cell face stretching^31^ followed by a change in the slope of the strain stress curve akin to ‘yielding’ at strains typically in the range 10–30% (Fig. 6). ‘Yielding’ is typically associated to the collapse of the cells and the mechanisms depend on the density, with stresses that can be up to few MPa for denser materials (Fig. 6d).

As expected, the elastic modulus increases with the density as represented in Fig. 6c with other carbon-based materials^8^,^32^,^33^,^34^. Interestingly, an empirical power law fitting results in an exponent significantly lower than those reported for other materials in the literature and even lower than 2. The deviation from the quadratic dependence and the increased weight of the linear term when using a a*ρ*^2^+b*ρ* fitting (Fig. 6c) suggest that the networks behaviour is closer to closed-cell cellular structures where the membrane stresses have a significant contribution to the elastic modulus^31^. However, it has to be also noted that the measurement of the Young’s modulus in compression, particularly at very low loads, can be fraught with higher errors as evidenced by the higher deviation in modulus values for low-density materials.

Denser rGO-CNs (typically with densities above 10 mg cm^−3^) or those that have been thermally treated at higher temperature reach a plateau after brittle collapse with visible micro-fracture events similar to those observed in the compression of elastic-brittle foams (Fig. 6b)^26^,^35^. However, and despite the micro-fractures, elastic-brittle networks can exhibit recoveries of up to 98% in the first compressive cycle after strains of 50%. This is similar to the behaviour of Ni microlattices that also exhibit damage and recovery during a compressive cycle^26^. The exceptions are the structures with densities >100 mg cm^−3^, which do not recover after compression that will be consistent with the extensive damage of the cell walls and edges. Lighter materials (densities below ~10 mg cm^−3^) recover up to 99% during unloading. After ‘yielding’, their stress/strain curve continues rising although the slope is significantly lower (Fig. 6a). This behaviour resembles more an elastomeric foam where collapse is determined by elastic buckling of the walls^31^.

Overall, the rGO-CNs are significantly more resistant than Ni microlattices of similar density and for the lightest networks, the stresses required to reach 50% strains are up to two orders of magnitude higher (Supplementary Fig. 8). The behaviour is very similar for all structures in subsequent cycles. The curves exhibit what can be described as a pseudohardening behaviour, in which the slope of the curve increases very fast with strain corresponding to significant densification with a large degree of recovery during unloading (typically between 99 and 97% for rGO-CNs with densities <100 mg cm^−3^). In general, a gradual degradation in the properties is always observed in the first four cycles, with the biggest drop always between cycles 1 and 2 but then the curves tend to stabilize (Supplementary Fig. 9a) with recoveries that can be up to 95% for the lightest foams after 10 cycles of 50% strain.

The capability of a material to absorb energy is key for several applications, especially in energy conservation systems for space and transportation between others. For these technologies, energy absorption and low density in high performance materials is extremely important for improved cost effectiveness. The energy loss coefficients (the ratio between the energy dissipated within the materials and the work done by compression during the first cycle) can be as high as 0.86 for the lighter materials. The energy absorption capability are among the highest found for foams surpassing the values of foam-like CNT films^36^ (~0.64), 14 mg cm^−3^ Ni micro lattices^26^ (0.77) and just >0.82 obtained for 5.1 mg cm^−3^ graphene elastomers^8^.

Furthermore, a good cycling performance is maintained, stabilizing the coefficient values at ~0.55 after the first four compression cycles (Supplementary Fig. 9a).

We can tune the energy absorption capability of the ultra-low density rGO-CNs exhibiting recoverable deformation by controlling their reduction degree. For example, the energy loss coefficient of 0.86 for rGO-CNs with a density of 6.1 mg cm^−3^ after reduction at 300 °C (Fig. 6a) can go to 0.51 for rGO-CNs of the same composition reduced at 1,000 °C (Supplementary Fig. 10). The higher reducing temperature leads to the formation of higher amounts of C sp^2^ bonding^24^. The highly crystallized rGO undergoes less damage through cycling and consequently dissipates less energy while maintaining an extremely good dimensional recovery.

The high levels of energy dissipation within the rGO-CNs can be attributed to a combination of mechanisms taking place at different length scales during compression. At the nanoscale, friction and Van der Waals attraction^8^ between rGO flakes during loading and unloading are two mechanisms responsible for the high energy loss coefficient during cycling. Specifically, the micro-roughness and organization of rGO flakes in the cell walls increases the contact area, leading to a high degree of friction between flakes while buckling and recovering. In addition to the above-described mechanism, the extra high levels of energy dissipation taking place during the first compression cycles is a result of micro rupturing and wrinkling in the cell walls that remain after the cycle (Supplementary Fig. 9b). These damage mechanisms contribute to the non-recoverable deformation and the decrease in mechanical properties upon cycling (Supplementary Fig. 9a). At the micro-to-macro scale, the materials’ cellular structure contributes to the high degree of recoverable deformation. Each cell wall can deform and recover differently from the stresses. Depending on the cell size, for example, some of them can present more deformation or fracture during buckling/bending than others. However, the predominantly undamaged cells can be recovered from the deformation leading to the macroscopic elastic behaviour of the rGO-CNs.

**Figure 7:**
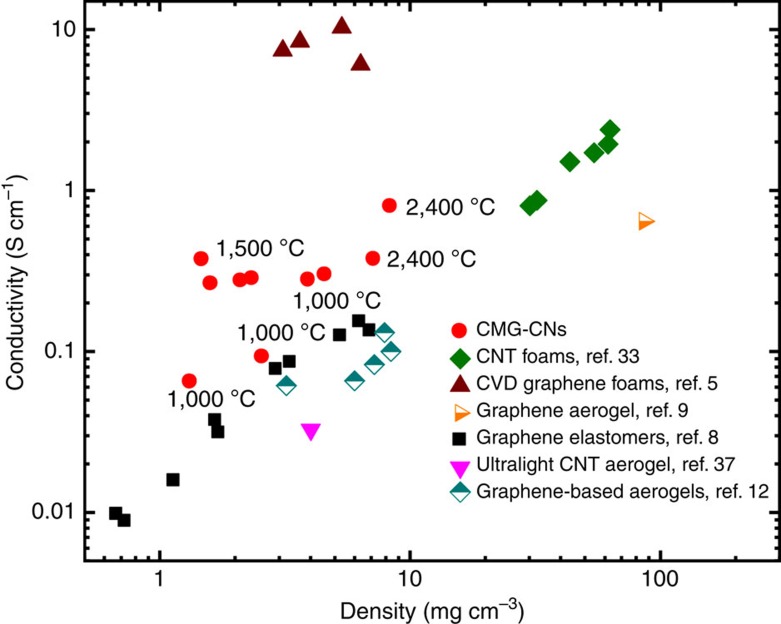
Electrical conductivity of rGO-CNs as a function of density together with several low-density carbon nanomaterials reported in the literature. The data correspond to rGO-CNs with different densities (produced with the addition of 0.3 or 1.2 wt% additives in the GO-sus) after being thermally treated in vacuum (graphite furnace) between 1,000 and 2,400 °C (annealing temperature identified in the data points).

The electrical conductivity of rGO-CNs is shown together with literature values for other 3D carbon nanomaterials^5^,^8^,^9^,^12^,^33^,^37^ (Fig. 7). We can tune the electrical conductivities of the rGO-CNs to very high levels; surpassing other 3D materials produced from GO^8^ or CNT aerogels^37^ with similar densities. The high crystallinity resulting from thermal reduction of CMG at temperatures above 1,000 °C (Fig. 4e) together with their high degree of interconnection in the cellular network leads to a highly conductive path. Although the conductivities are still below those of graphene chemical vapour deposition foams^5^, the electrical conductivity of an ultralight rGO-CN of only 1.5 mg cm^−3^ (reduced at 1,500 °C), reaches 0.4 S cm^−1^ which is one order of magnitude higher than that of a graphene elastomer with similar density.

### Absorption of organics

**Figure 8:**
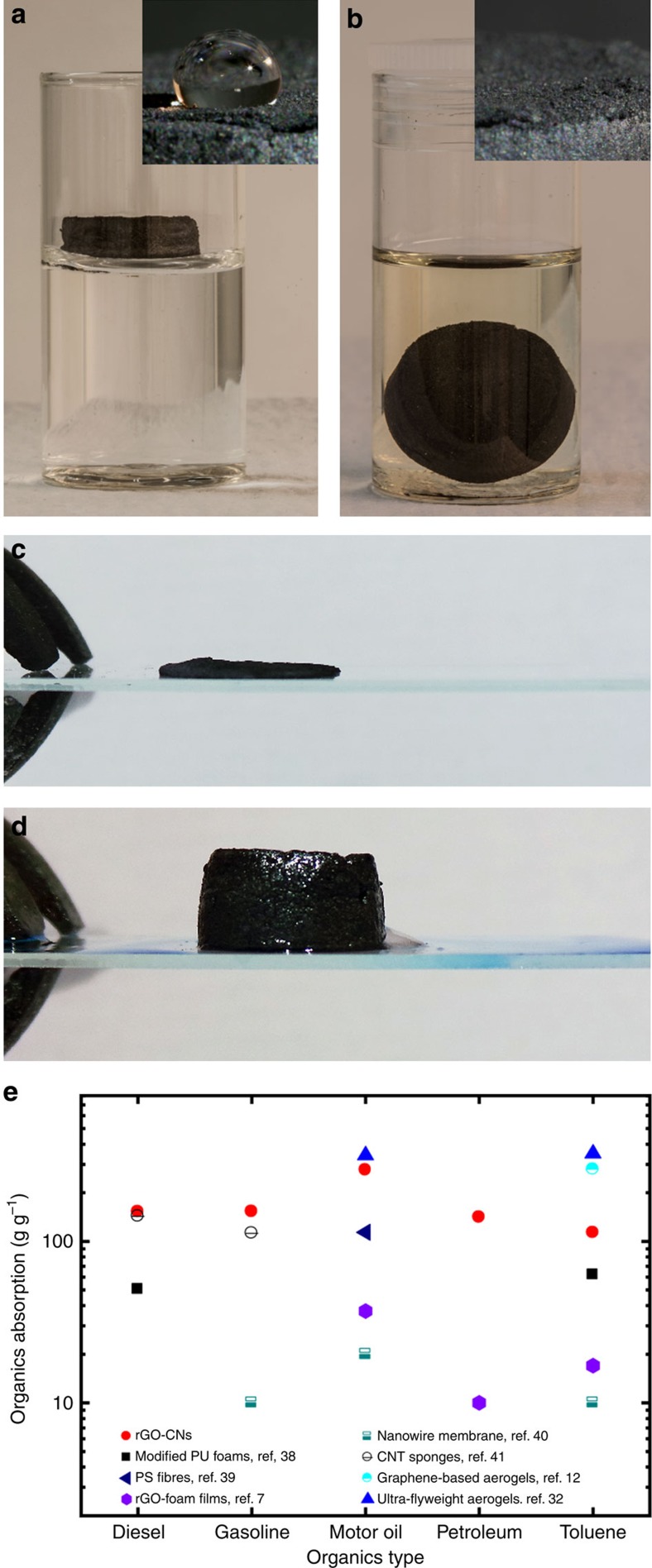
rGO-CNs capability for organics absorption, wetting behaviour and recycling approach. (**a**) The rGO-CN floats when in contact with water due to its superhydrophobic properties. Water droplet forms a contact angle of 114° with the rGO-CN surface (insert). (**b**) rGO-CN rapidly absorbs gasoline filling the highly porous structure with the solvent resulting on its immersion in the gasoline vial. In the insert, gasoline trace left after infiltration in the rGO-CN. (**c**,**d**) Recycling approach for rGO-CNs absorbers. After each absorption cycle, the oil phase can be ‘squeezed out’ of the rGO cellular absorber by compressing it (**c**) and the compressed structure can be directly re-utilized by immersing it in the oil phase again. The absorber immediately expands to its original shape by the absorption of the oil phase within its structure (**d**) (details in Supplementary Movie). (**e**) Organics absorption (g g^−1^) of rGO-CNs in comparison with several absorbers reported in literature for different organic solvents and oils. The rGO cellular absorbers tested (4–4.5 mg cm^−3^) were produced with 1.2 wt% additives in GO-sus (0.65 wt% GO) and thermally treated at 1,000 °C in Ar/H_2_ atmosphere.

The rGO-CNs exhibit the perfect characteristics of an organics (organic solvents and oils) absorbent: ultra-low density, extremely high porosity, superhydrophobicity (Fig. 8a), very good wetting for organics (Fig. 8b), strength and good dimensional recovery. While the rGO cellular network floats when immersed in a vial with water, it efficiently absorbs the organic solvents.

We tested the absorbing capability of the rGO-CN with 99.8% porosity (4.3 mg cm^−3^ density) with oils and organic solvents (Fig. 8e) and compared with different materials from the literature, including polymeric foams^38^, organic fibres^39^, rGO-foam films^7^, nanowire membranes^40^, CNT sponges^41^, graphene-based aerogels^12^ and ultra-flyweight aerogels^32^. The rGO-CNs are among the best absorbers with organics intake reaching 113 to 276 times their own weight. The absorption capability will also strongly depend on the absorber density. In this way, a lighter rGO-CN of 1.5 mg cm^−3^ can increase its absorption capability for motor oil from 276 g g^−1^ to values as high as 605 g g^−1^.

Due to its mechanical and chemical stability, the rGO-CNs maintain the cellular network integrity after exposure to the organics for several weeks. The oil phase can be ‘squeezed out’ of the rGO cellular absorber by compressing it flat above 95% of its initial height (Fig. 8c) and the compressed structure can be directly re-utilized by immersing it in the oil phase again (even before it recovers the shape). The absorber immediately expands to its original shape by the absorption of the oil phase within its structure (Fig. 8d). This process can be repeated over several cycles (at least six were tested for each structure) while maintaining the adsorption capability to levels >95% (Supplementary Movie). This recycling approach is very straightforward, simple and uses the rGO cellular networks as a compressible/expandable absorber making them promising candidates as organics absorbers, filters and membranes for environmental applications.

## Discussion

In summary, we have developed a mesoscale self-assembly strategy for the highly efficient fabrication of CMG cellular networks. This approach allows the manipulation of the structure at multiple levels from the densities (over two orders of magnitude from 1 to 200 mg cm^−3^), cell shape (polyhedral to spherical) and sizes (~7 to over 60 μm) at the micro-level to the cell walls topography, porosity and chemistry at the micro-to-nano-level. As a result it is possible to tune properties like, surface area, elasticity, specific strength, energy loss coefficient and conductivity. Further, due to the intrinsic flexibility of emulsions, it is possible to extrude CMG wires with cellular architectures showing promise for the fabrication of complex structures at the macroscale. This opens up new opportunities to explore applications in numerous fields like in energy damping, compression tolerant supercapacitors or catalyzers or any application where separation, absorption or filtration is required. In special, we have shown that due to the ultra-low density, ultra-high porosity and wettability to organics, the rGO-CNs are promising candidates as absorbers for environmental applications, such as in oil spill clean-up.

## Methods

### Fabrication of CMG-CNs

The general processing route for the rGO-CNs is displayed in Fig. 1e. GO solutions were prepared using the modified Hummers method^42^ and subsequently freeze-dried (Freezone 4.5, Labconco Corporation) to obtain GO flakes. GO flakes were dispersed in water or aqueous solutions containing organic additives (PVA:sucrose in a 1:1 fixed ratio) using an ultrasonic tip (UP200S, Hielscher) for 5 min to obtain homogeneous 0.65 wt% (6.5 mg ml^−1^) GO-sus. The aqueous GO-sus were then emulsified with a hydrophobic phase (toluene) by hand shaking. The two phases (GO-sus and toluene) formed a homogeneous GO-em containing up to 75 vol% of the internal phase toluene droplets. The GO-em was casted into cylindrical Teflon moulds and unidirectionally frozen at 10 K min^−1^ in a house-built freeze caster to promote the formation of ice growth within the continuous phase surrounding the droplets. Bulk GO-CNs (with cylindrical shape of~18 or 20 mm in diameter and 9 or 15 mm in height) with densities between 3 and 15 mg cm^−3^ were obtained by freeze drying (Freezone 4.5, Labconco Corporation) the frozen GO-em produced from GO-sus containing 0 to 5 wt% organic additives, respectively. The rGO-CNs were thermal treated between 300 and 1,000 °C in 10% H_2_/90% Ar atmosphere inside a tubular oven (Carbolite Furnaces) and between 1,000 and 2,400 °C in a graphite furnace (FTC model HP W 25) under high vacuum (25–60 Pa).

### Characterization of CMG-CNs

The microstructural architecture of CMG-CNs was analyzed via scanning electron microscopy (LEO Gemini 1525, operated at 5 kV) and STEM (FEI Titan 80–300 S/TEM, operated at 300 kV). STEM high-angle annular dark field images were acquired with a collection of semi-angles >101 mrad. Cell sizes were measured from scanning electron microscopy images using the linear intercept method (Linear Intercept, TU Darmstadt). The average cell size (d_50_) was obtained from cumulative size distribution curves. Electron energy loss spectroscopy acquisitions were performed in STEM mode with an incident angle *α*=10 mrad and collection angle *β*=7 mrad. The spatial and energy resolutions of the microscope under the experimental conditions were ~0.5 nm and ~0.7 eV (defined as the full width at half maximum of the zero-loss peak), respectively. Raman measurements were carried out with a spectrometer (Renishaw RM2000 CCD) using a 514 nm laser excitation, laser power of 0.5 mW and 10 s integration time. The laser was focused onto the sample using a 50 times short working distance objective. Several spectra were collected from random locations on each sample. XPS spectra were collected using Al Kα X-ray source.

The CMG-CNs specific surface area was determined with an Autosorb-6B (Quanta Chrome) using the Brunauer–Emmett–Teller method.

To ensure reproducibility and reliability, the measurements were performed on >5 mg of CMG-CNs, which were placed inside plastic capsules closed with filter paper. Using closed capsules ensured an accurate weighing of the samples and avoided the extremely light foams from getting suctioned during vacuum. The reliability was confirmed with measurements with different weights. CMG-CNs were degassed in the Autosorb degasser (Quanta Chrome) under 0.03 mbar for at least 24 h before the nitrogen adsorption. The nitrogen adsorption isotherm measurements were performed at relative pressures (P/P_0_) between 0.01 and 1 at a bath temperature of 77 K with 40 points for adsorption and 39 points for the desorption. Cylindrical samples (~20 mm in diameter and 10 mm in height) were used for measuring density, mechanical testing and electrical conductivity measurements. The densities of the CMG-CNs samples *ρ* were determined by measuring their dimensions with a standard caliper and their mass with a 0.01 mg accuracy balance. The porosity P was estimated considering the density of the rGO-CNs cell walls *ρ*_*c*_ as 2.2 g cm^−3^ (ref. 43) with the following equation: P=1−(*ρ*/*ρ*_*c*_) × 100. Repeated compressive load and unload tests were performed in a universal mechanical testing machine (Z2.5, Zwick Roell, Germany). The rGO-CNs cylindrical samples were submitted to 10 compressive cycles of 50% maximum strain, using a 2 kN load cell, in position controlled mode at loading and unloading speed of 0.01 mm s^−1^. The electrical conductivity of rGO-CNs was measured using the four-point method.

### Organics absorption test on rGO-CNs

The absorption capability of rGO-CNs was evaluated by immersing the absorbent samples on a selection of organic solvents and oils and measuring the weight uptake with the following equation: Organics absorption (g g^−1^)=(wt_f_−wt_0_)/wt_0_, where wt_0_ corresponds to the initial dried weight of the rGO-CN absorbent and wt_f_ to the weight of the sample infiltrated with the organics.

## Author contributions

S.B. designed the processing approach, directed the experiments and wrote the manuscript with input from the other authors. E.S. with the contribution of C.M. mentored the work and revised the manuscript. F.M.P. and P.d.V.P. prepared and characterized the samples. F.M.P. and S.B. analysed mechanical and electrical performance and F.M.P. performed organics absorption tests. S.B., F.M.P. and P.d.V.P. performed SEM. N.N. performed STEM and electron energy loss spectroscopy (EELS) characterizations. S.E. performed nitrogen adsorption tests. C.M. directed the RAMAN and the XPS characterizations. C.M. and R.C.M. performed Raman and S.A. XPS characterization. S.B. and E.G.-T. developed the CMG-CN wires and performed contact angle measurements on GO films.

## Additional information

**How to cite this article:** Barg, S. *et al.* Mesoscale assembly of chemically modified graphene into complex cellular networks. *Nat. Commun.* 5:4328 doi: 10.1038/ncomms5328 (2014).

## Supplementary Material

Supplementary Figures Supplementary Figures 1-10

Supplementary Movie 1 rGO cellular networks as a compressible/expandable organics absorber. Gasoline (in blue) can be "squeezed out" of the rGO cellular absorber by compressing it and the compressed structure can be directly re-utilized by immersing it in the oil phase again. The absorber immediately expands to its original shape by the absorption of the oil phase within its structure. This process can be repeated over several cycles.
