# Supplementary material for: Mesoscale assembly of chemically modified graphene into complex cellular networks
Source: Nat Commun. 2014 Jul 7;5:4328. doi: 10.1038/ncomms5328 (PMC4102120; doi:10.1038/ncomms5328)
Supplement: Supplementary Figures — 1-10 [file ncomms5328-s1.pdf]

Supplementary Figures

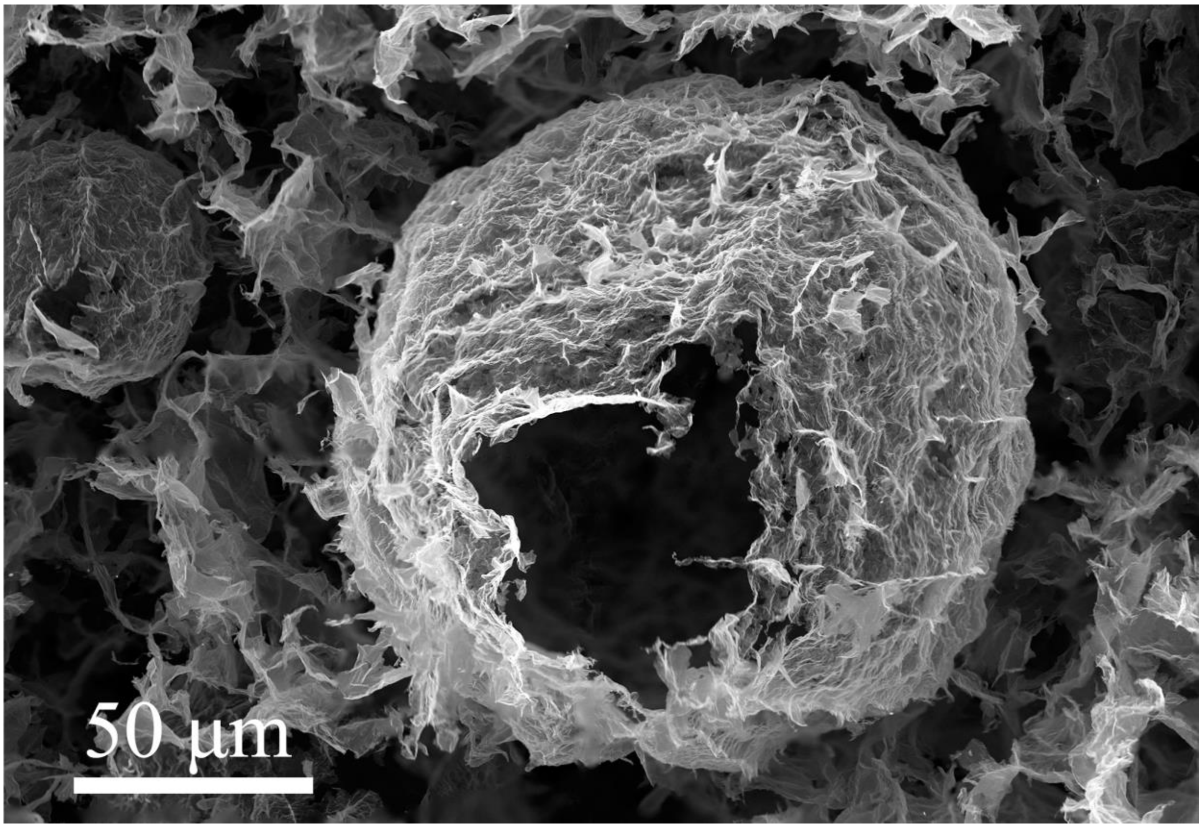

Supplementary Figure 1: SEM image of a spherically shaped closed cell encapsulated by CMG flakes. CMG segregates to the oil/water interface during emulsification, stabilizing spherically shaped emulsion oil droplets that after freeze drying will lead to cell covered by CMG flakes in the CMG-CNs.

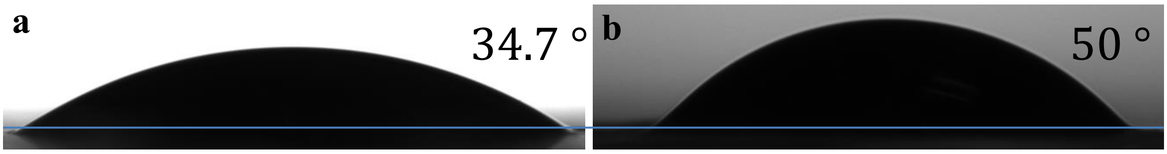

Supplementary Figure 2: Contact angle of water on GO films without (a) and with the addition of PVA (b). PVA molecules adsorbed on GO flakes reduce the contact angle of water by 15 ° indicating a change of wettability for GO.

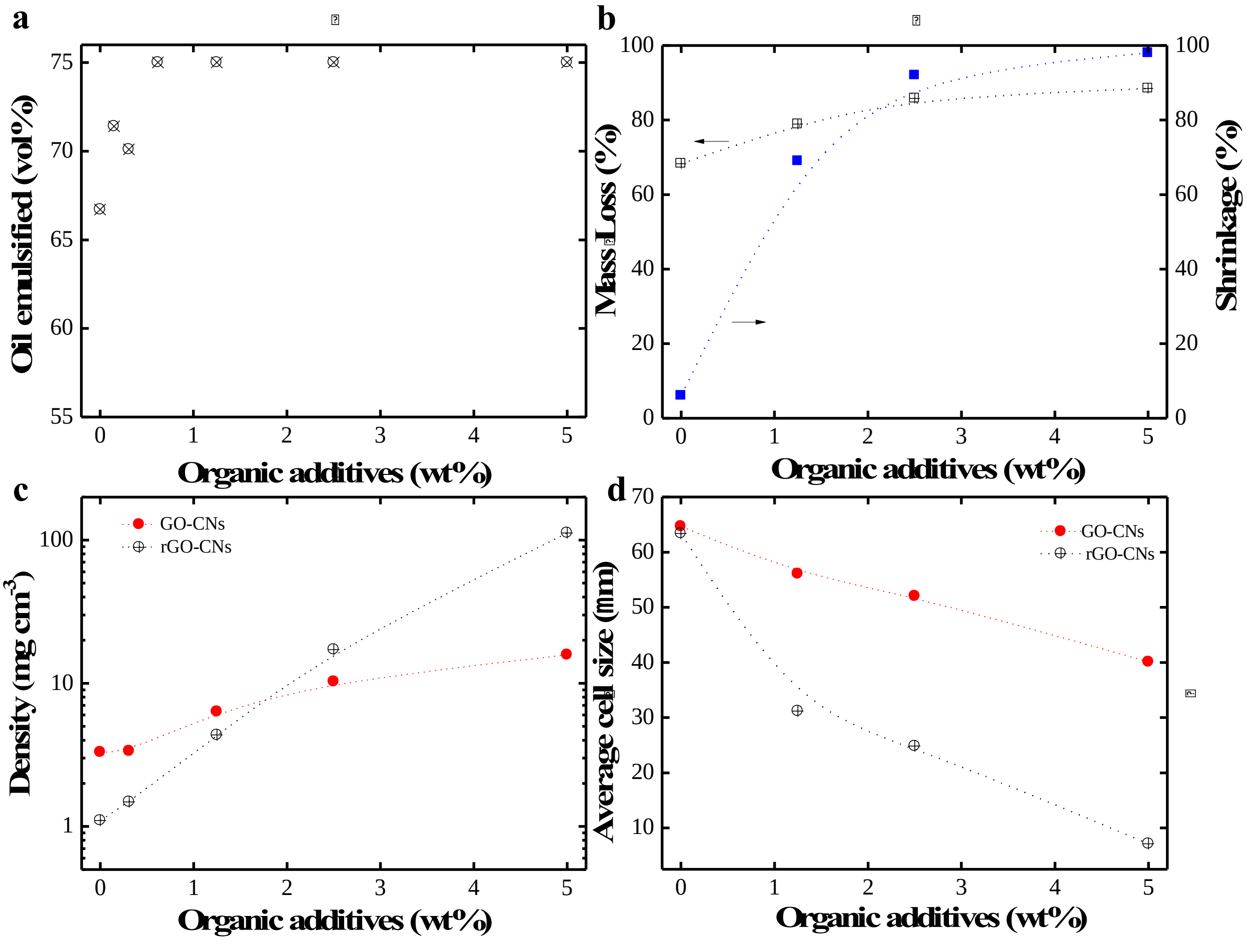

Supplementary Figure 3: Processing and structural characterization of GO- and rGO-CNs as a function of additives. a. Maximum amount of oil emulsified in the GO-sus in order to get GO-em; **b.** mass loss and shrinkage of GO-CNs upon thermal annealing at 1000 °C in Ar/H_2 atmosphere; **c.** Density and **d.** Average cell size (d_{50}) of the resulted GO and rGO-CNs. The organic additives (PVA: sucrose in 1:1 wt%) are added to the GO-sus with 0.65wt% GO.

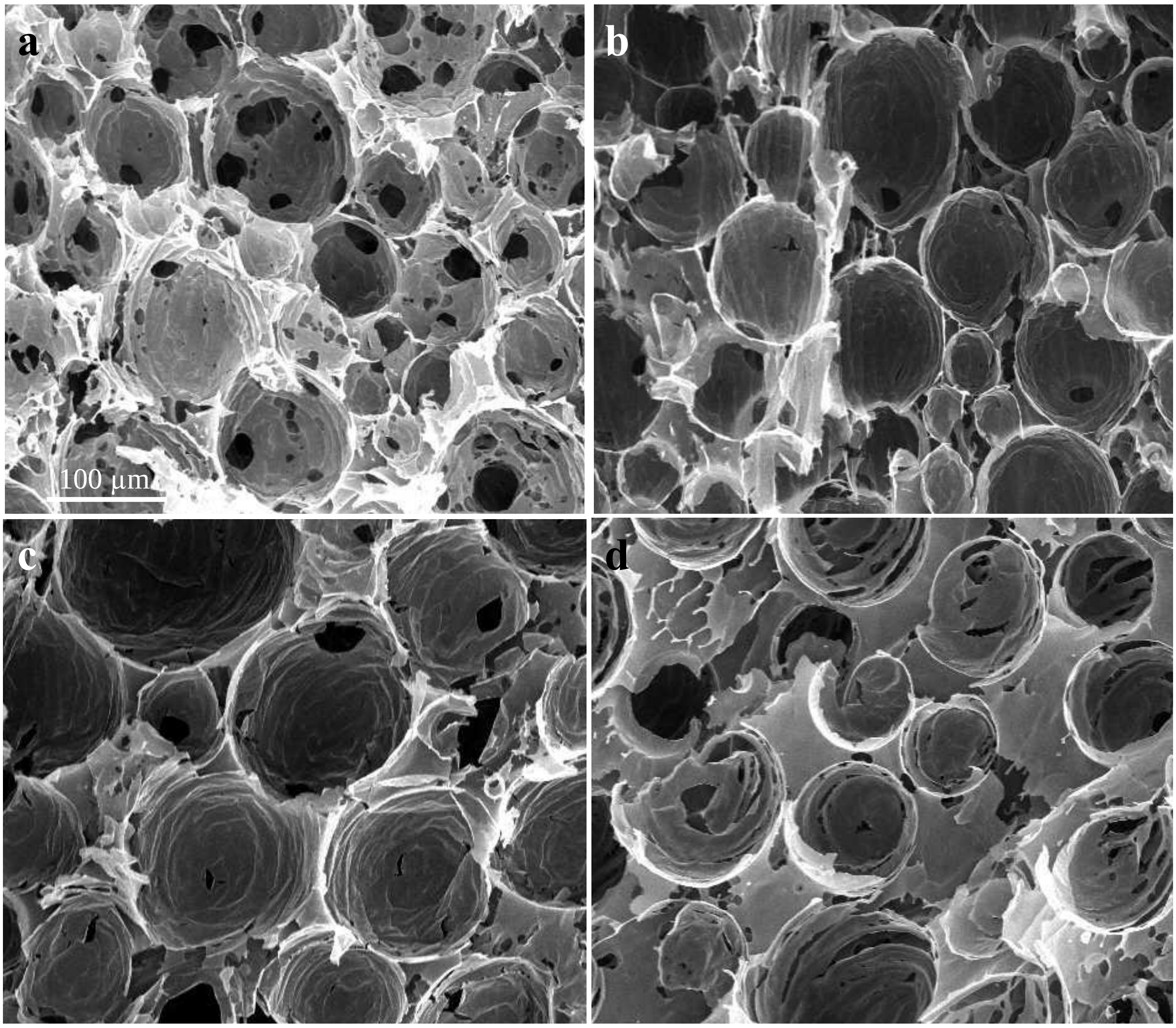

Supplementary Figure 4: **Effect of organic additives sucrose and PVA on the CMG-CN cell wall topography.** GO-CNs produced from suspensions containing 0.65 wt% GO and 1.2 wt% organics, with different proportions of PVA:sucrose: 3:1 (**a**), 1:1 (**b**) and 1:3 (**c**) or with 5 wt% organics (sucrose) (**d**). Scale bar for all figures in **a.**

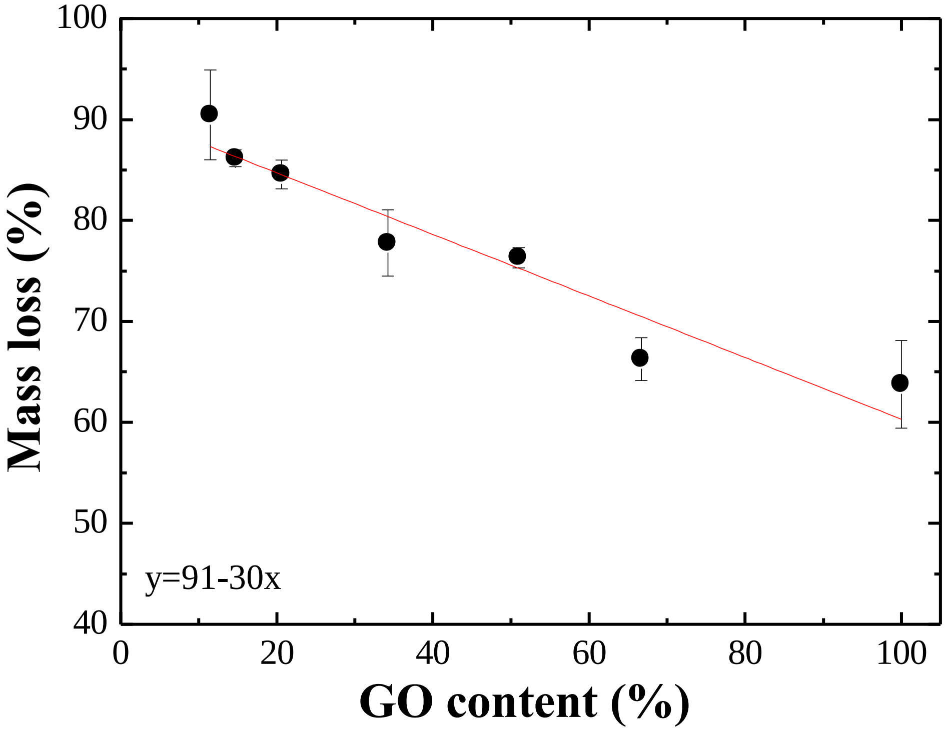

Supplementary Figure 5: Mass loss during reduction at 1000 °C in Ar/H_2 atmosphere versus proportion of GO in the GO-CNs. GO content with regards to total amount of solids, i.e. GO and organic additives. The linear fit indicates that 0.91% of the binder is eliminated during the thermal treatment.

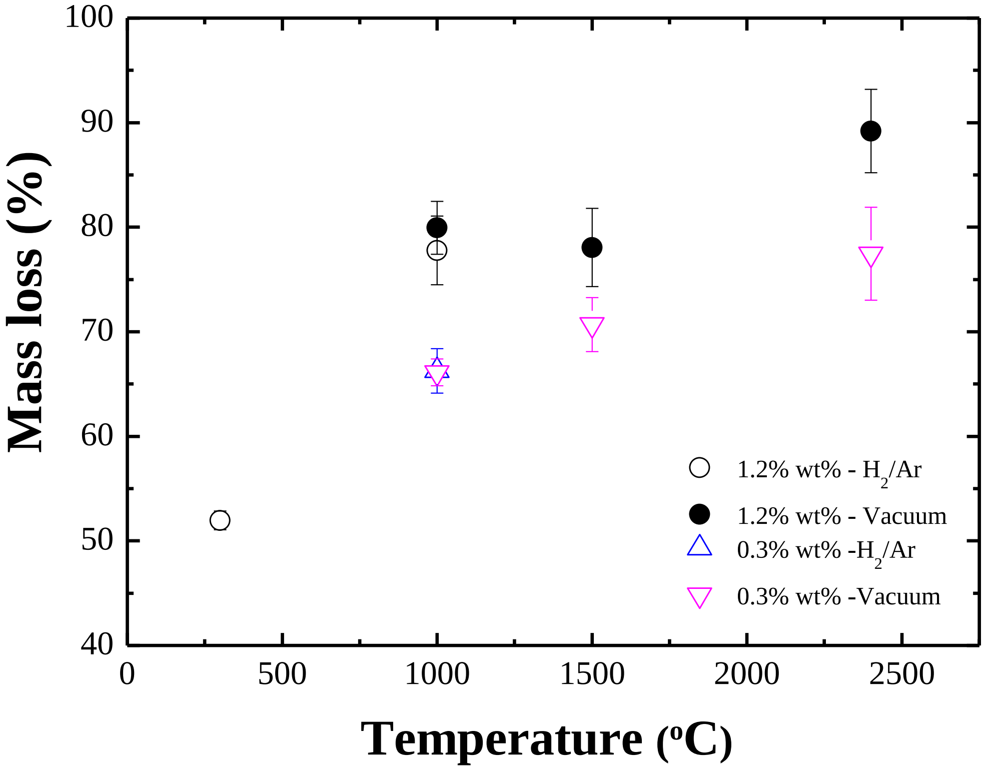

Supplementary Figure 6: Proportion of mass loss during thermal treatment at different temperatures for CMG-CNs produced with 0.3 and 1.2 wt% binders.

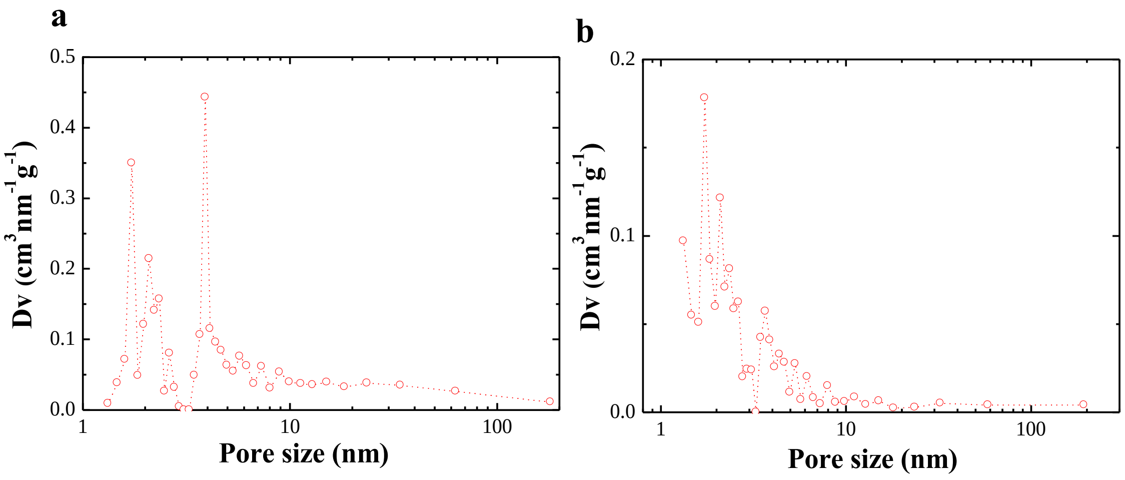

Supplementary Figure 7: Differential pore volume distribution on rGO-CNs obtained by Barret-Joyner-Halenda (BJH) method applied to desorption isotherms. a. Additive-free rGO-CNs. The peak at 4 nm results from nitrogen cavitation during the desorption; b. rGO-CNs produced with 1.2 wt% additives.

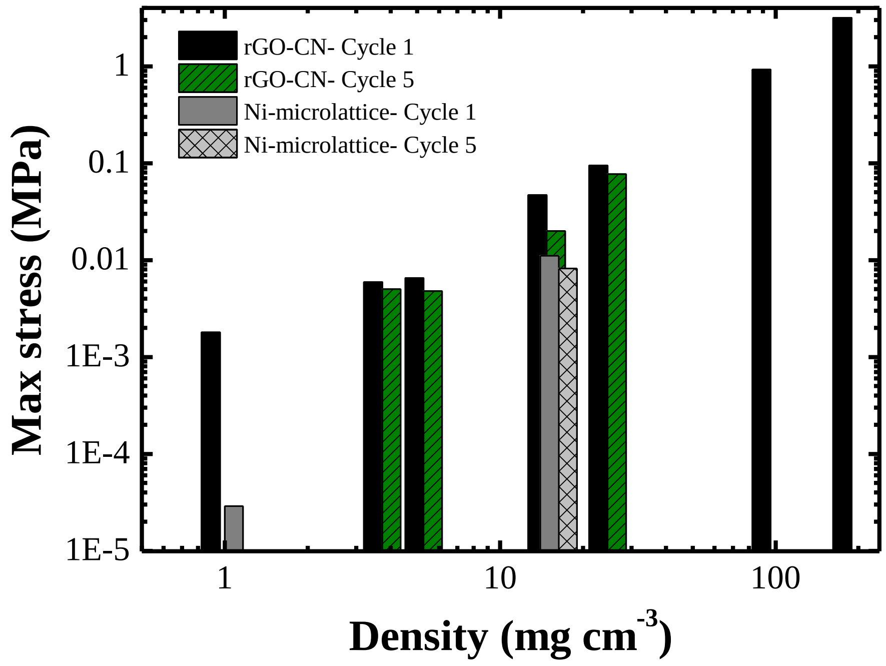

Supplementary Figure 8: Maximum stress at 50% compression at cycle one and five (when there is recoverable deformation) for rGO-CNs and Nickel microlattice[26] as a function of material's density. rGO-CNs of different densities produced with 0 to 5 wt% additives and thermal annealed at 300 and 1000 °C.

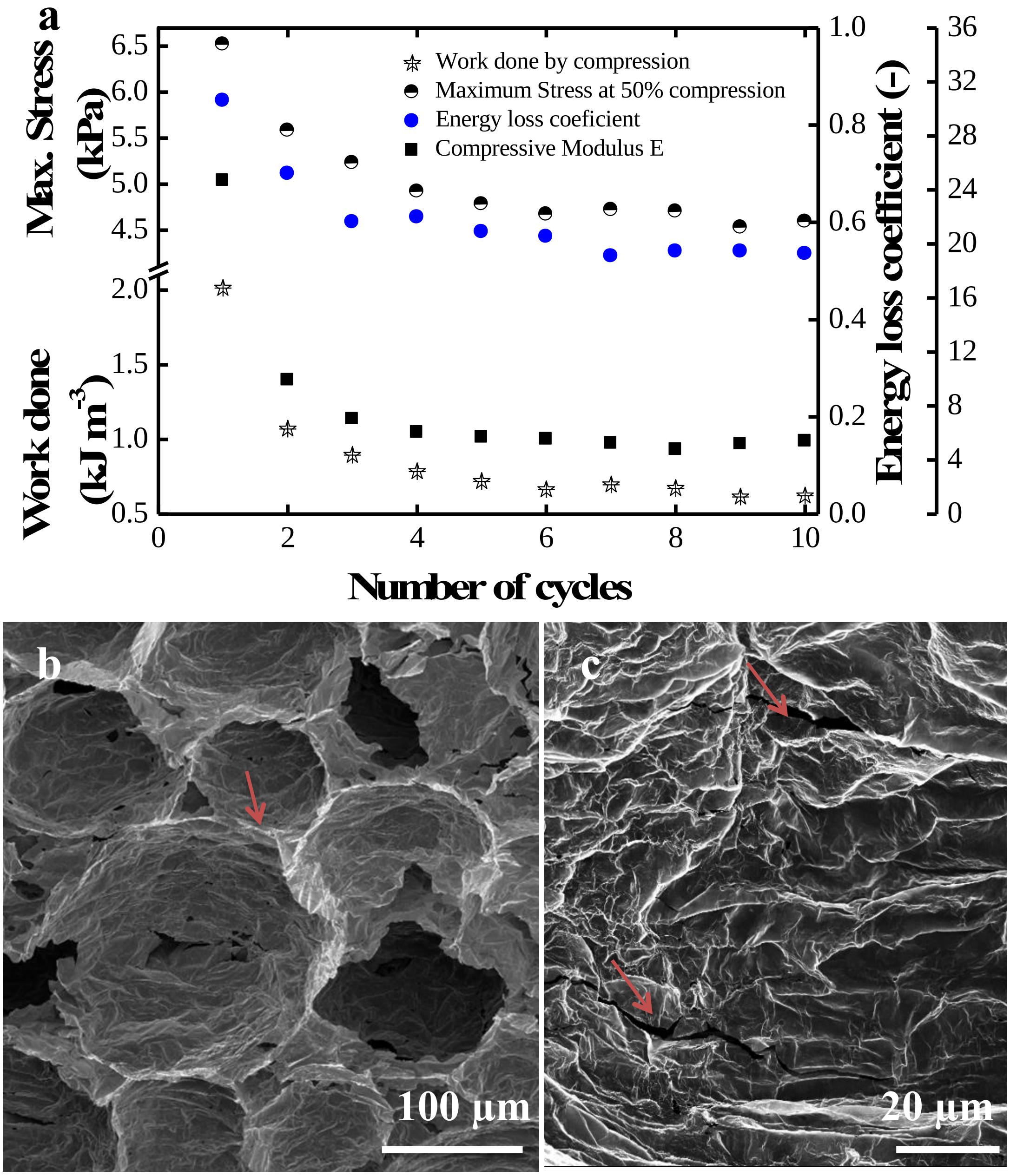

Supplementary Figure 9: Cycling performance of rGO-CNs (6.1 mg cm^{-3}) presenting recoverable deformation. a. History of maximum stress, work done by compression, energy loss coefficient and compressive modulus E for ten compression cycles at 50% strain. **b-c.** SEM images of recovered cellular network after it underwent ten compressive cycles**.** A microstructural overview showing the recovered cells and intact struts (indicated with the arrow) efficiently supporting the adjacent cells is shown in **b**. In **c.** rupture paths within the cell walls can be identified (arrows). They tend to be oriented towards the roughness (wrinkles) present on the cell wall. rGO-CN produced with 1.2 wt% additives and thermal annealed at 300 °C.

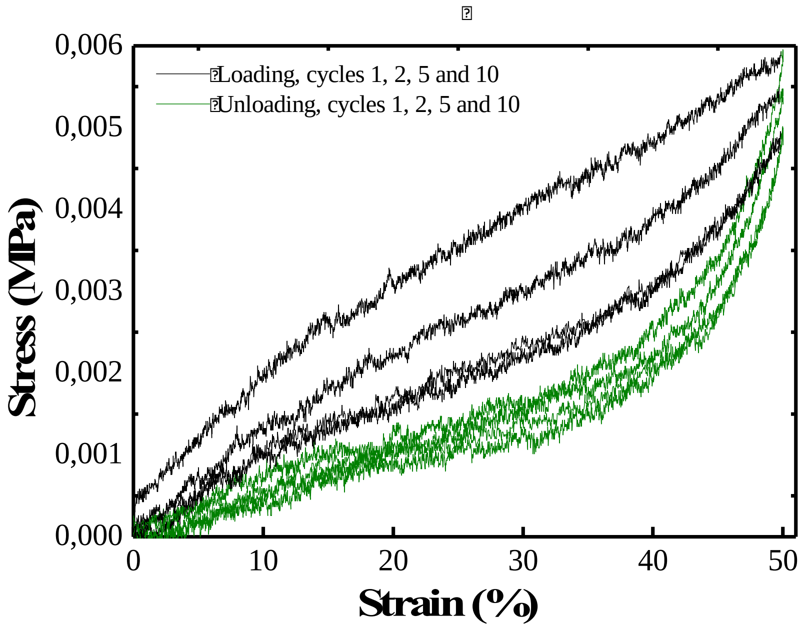

Supplementary Figure 10: Multicycle compression stress-strain curves of rGO-CN presenting recoverable deformation of 99 and 97 % after the first and tenth compression cycles. Loading-unloading curves of cycles 1, 2, 5 and 10 for an rGO-CN with 4.3 mg cm^{-3}. rGO-CN produced by the addition of 1.2 wt% additives in the GO-sus and annealed at 950 °C.
